# A Study Protocol of Realist Evaluation of Palliative Home Care Program for Non-Cancer Patients in Singapore

**DOI:** 10.5334/ijic.6497

**Published:** 2022-10-20

**Authors:** Milawaty Nurjono, Karen Liaw, Angel Lee, Hubertus Johannes Maria Vrijhoef, Lip Hoe Koh, Melissa Tan, Foong Ling Ng, Hong Choon Oh

**Affiliations:** 1 Health Services Research, Changi General Hospital, Singapore; 2 Centre for Population Health Research & Implementation, Singapore Health Services Pte Ltd, Singapore; 3 St. Andrew’s Community Hospital, Singapore; 4 Panaxea, Amsterdam, The Netherlands; 5 Geriatric Medicine, Changi General Hospital, Singapore; 6 ILTC Integration, Changi General Hospital, Singapore; 7 Health Services & Systems Research, Duke-NUS Medical School, Singapore

**Keywords:** palliative care, home care services, realist evaluation, non-cancer

## Abstract

**Introduction:**

Violet Program (ViP) was developed *to* address the current home palliative service gap for individuals with life limiting non-cancer conditions residing in the Eastern part of Singapore. While its basic principles and processes have been planned and implemented, how ViP works, for whom and in what circumstances are not yet well understood. Therefore, we propose for a realist evaluation (RE) – a theory-based evaluation, to address the current knowledge gaps. Evaluation findings may guide, support further development and broader uptake of ViP.

**Methods and Analysis:**

This study will be conducted in three phases: 1. development of initial program theory (IPT), 2. testing of programme theory, and 3. refinement of IPT. First, IPT will be elicited through review of programme documents, scoping review of reviews and in-depth interviews with stakeholders involved in the conceptualization of ViP. Then, a convergent mixed method study will be conducted to assess contexts (C), mechanisms (M) and outcomes (O) to test the IPT through interviews with stakeholders, surveys and analysis of program and administrative databases. Based on findings gathered and through consultation with respective stakeholders, IPT will be refined to highlight what works (outcomes), how (mechanisms) and for whom under what conditions (contexts).

## Introduction

### Background

Historically, palliative care (PC) focused largely on patients with cancer. However, as the number of deaths attributed to non-cancer causes exceed cancer-related deaths [1] with patients experiencing similar distressing symptoms negatively affecting their quality of life [2,3], there is a growing realization of the importance of extending PC to non-cancer conditions.

PC provided to terminally ill non-cancer patients has been found to be effective in reducing acute care utilization [4] and improving quality of life. Nevertheless, compared to those with cancer, utilization of PC among patients with non-cancer diseases is much lower and later in their disease trajectory [3,5]. Uncertainty caused by unpredictable illness trajectory and low accuracy in the prognostication of non-cancer conditions are key barriers [6]. Lack of knowledge regarding the needs of end-stage non-cancer patients and their families and limited PC training for healthcare professionals are also associated with low use of and late referral to PC [3]. As non-cancer patients often outlive their prognosis and end up needing PC for an extended period of time, longer term sustainability is a concern [5]. Many specialist PC services are hesitant to take in such cases, resulting in high numbers of unmet needs.

Similar to other developed countries, there is a growing need for PC for a range of life limiting illnesses in Singapore [7]. Disproportionately higher deaths attributed to non-cancer causes highlight the need for PC beyond those with cancers [8]. Thus, enhancement in the provision of PC is prioritized at the national level [9]. Provision of home-based PC increases the chance of dying at home [10,11], improves symptom control [11] and quality of life [12], reduces hospital utilization [12,13] and healthcare cost in non-cancer conditions.

Traditionally, in Singapore, PC is provided to patients suffering (Ministry of Health Singapore, n.d.) from cancers in hospitals, hospices and at home [14,15] and significantly lower proportion of individuals with non-cancer conditions were referred and received home PC (unpublished data). As studies [16,17] have consistently demonstrated that non-cancer patients have similar, if not more, needs that may benefit from PC, this highlights an apparent service gap.

Contrasting needs, goals and care experiences among those with advanced illnesses nearing the end of life limits generalizability of existing cancer-based PC models [18]. This suggests that tailored PC programs are required for distinct disease groups. Furthermore, as home PC has been primarily provided by specialist home hospice providers based on individual’s prognosis [19], sustainability is a real concern due to limited number of such specialists. When patients outlive their prognoses or “stabilize”, home hospice services are often compelled to hand-off care to allow enrolment of more symptomatic patients. Not only is such a model unsustainable, it also lacks continuity of care.

There are compelling reasons for a new model of home PC to cater to the unmet yet growing needs of those with terminal non-cancer diseases. The World Health Organization (WHO) and National Strategy for Palliative Care [14,20] recommend for a well-integrated program which leverages on multidisciplinary collaboration and input of providers from different care settings.

### The Intervention: Violet Program

The Violet Program (ViP) was developed as a collaboration between Changi General Hospital (CGH) and St. Andrew’s Community Hospital (SACH) to provide home-based PC to non-cancer patients residing in the Eastern region of Singapore. VIP aims to (i) reduce unnecessary acute hospital utilization (ii) improve health outcomes (iii) reduce caregiver burden through caregiver support (iv) honour patient preferences better through integrated care while (v) keeping healthcare cost affordable.

CGH is a 1,000-bedder acute care hospital with a consult-based PC team who also runs an outpatient PC clinic. SACH is a step-down care rehab and subacute facility that also houses a 24-bedder inpatient palliative ward and home care services. ViP is an integrated program involving specialists from CGH PC team and SACH home palliative supporting the SACH generalist home medical and home nursing services. The specialist team is a multidisciplinary team comprising of doctors, nurses, medical social workers and pastoral care trained in PC with input from other allied health professionals (physiotherapists, occupational therapists, speech therapists) while the generalist team are doctors and nurses trained in care of chronic illnesses with exposure to end of life care issues. Using a common electronic platform, the specialist team provides care for patient and families with more complex care needs, e.g. symptom control requiring frequent medication titration, subcutaneous medication infusion, unstable symptoms or high psychological needs requiring frequent touch points. The generalist team provides basic PC by engaging patient and family in advance care planning (ACP), education on the dying trajectory, managing non-complex symptoms, providing caregiving training and identifying patients with escalating PC needs requiring specialist intervention. The generalists have lesser touch points of between 3–6 months unless a regular nursing procedure is needed, e.g. change of nasogastric tube, indwelling urine catheter, wound dressing. As such, the specialists also provide ad-hoc rapid response service for patients managed by generalists when acute events occur. The program provides a 24 hours service. An on-call phone number is given on enrolment and patient/Next-of-Kin is briefed on the use of on-call service. On-call number is manned by a PC nurse or doctor with a PC consultant as 2^nd^ line support. Advice over the phone or urgent home visits are done depending on assessment of the situation by the on-call staff. Patients who have stable PC needs over a period of time have their care de-escalated to the generalist team till their care needs next peak.

**Figure 1:**
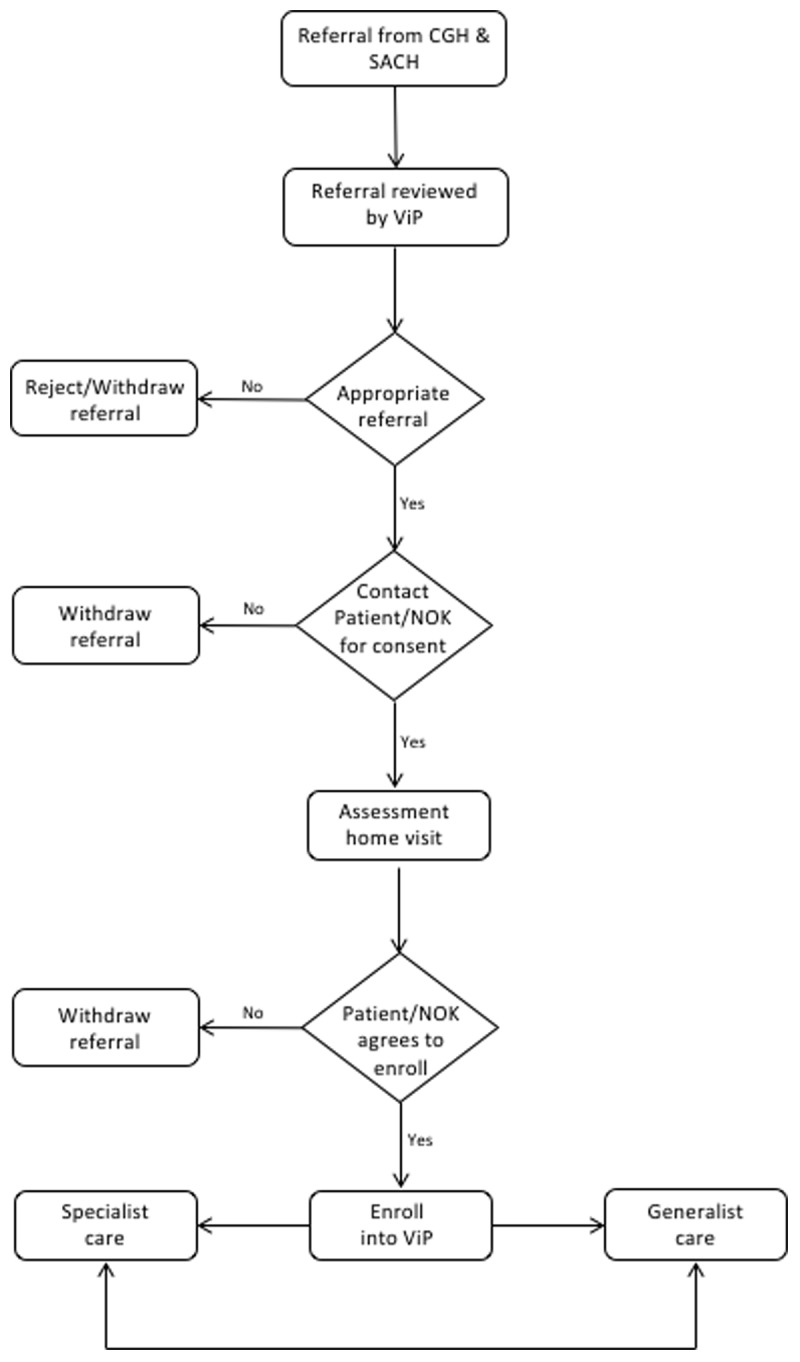
Recruitment workflow of ViP.

As of May 2022, majority of referrals came from CGH and SACH, with other organizations contributing only 7.5% of total referrals. Patients are identified in the inpatient, specialist clinic and home setting based on a pre-determined selection criterion. Pre-assessment by the PC team of the organizations is not required. In brief, the referral criterion includes (i) dementia (at least FAST 7), (ii) Chronic Obstructive Pulmonary Disease GOLD D, irreversible chronic lung diseases, (iii) heart failure (NYHA III or IV), or (iv) renal failure (eGFR <10 ml/min per 1.73 m^2^, not for dialysis) with prognosis of <12 months. Other forms of frailty, e.g. stroke or neurodegenerative illnesses, with prognosis <6 months are also accepted. This admission criterion is based on guidance laid down by Ministry of Health and Central Provident Fund Board who oversees the use of Medisave, a national medical savings scheme. Patients who do not fall within these criteria but assessed to have PC needs are discussed on an ad-hoc basis with ViP. Referrals are vetted by ViP doctors and suitable referrals are contacted and an assessment visit date is scheduled. Patients are enrolled into the program once the service agreement is signed. Figure 1 describes the recruitment process of ViP.

After enrolment, care is then stratified according to needs based on Palliative Care Outcomes Collaboration (PCOC) assessment [21]. Patients of higher acuity or requiring closer monitoring will be cared for by the specialists. Patients deemed more stable will be serviced by the generalists with ad-hoc visits by the specialists to manage acute deteriorations. For every patient enrolled, an individualized care plan is developed, revisited regularly and executed collaboratively by healthcare providers and patients/carers. Patients are contacted at least once a month via phone call, video call or home visit. Frequency of contact is guided by PCOC assessment at each contact. Weekly multidisciplinary team meetings are held to discuss new, complex cases and to review mortalities as a formal debrief and to identify bereavement needs. Allied health, community-based service providers or lay extenders are engaged to provide an additional layer of support for patient or caregiver depending on patient/carers’ needs. Service is provided from enrolment until (i) the patient passes away and bereavement support is no longer required, (ii) patient’s condition improves and/or stabilizes such that the patient is no longer deemed to suffer from a terminal illness and home PC support is not required, (iii) patient is admitted to an inpatient hospice for terminal care or admitted to the acute hospital for more than three months, (iv) patient or family requests to withdraw from the service and (v) patient moves out of the zone of coverage.

### Problem Statement

ViP recruited its first patient in December 2020 and is in its infancy. While the general program design, processes and guiding principles were drafted by the ViP team with inputs from policy makers, it is not well understood “for whom” the planned program will be suitable and “how” ViP will work and be sustainable in the local context. To the best of our knowledge, ViP is one of the first integrated home PC model for non-cancer patients in Singapore involving an acute hospital, home medical and nursing teams and an inpatient hospice hence there are limited relevant context-specific insights from which ViP can learn from. As such, a rigorous and practical evaluation strategy to address the existing knowledge gaps is warranted. Findings from such evaluation will provide insights to support the implementation and further development of ViP and similar programs in Singapore and beyond.

Commonly used evaluation design, Randomised Controlled trial (RCT) design, which focuses solely on outcomes evaluation is rigid when evaluating a complex intervention. It is unsuitable for evolving interventions and rarely adequately or even explicitly address the context-specific drivers behind any outcomes and their relationship to the underlying program theory [22]. This makes it difficult to interpret their findings for improvement purposes due to its limited relevance in reality. [23].

### Realist Evaluation

Realist evaluation (RE) is a more suitable approach for the evaluation of ViP. RE seeks to clarify implementation processes by providing a more explicit and in-depth understanding of the influence of existing and evolving contextual factors and mechanisms driving an intervention [24,25]. Furthermore, RE is able to accommodate inevitable changes surrounding intervention implemented in real-world settings.

The realist lens purports that an intervention is made of active theories that can only achieve successful outcomes if appropriate ideas are applied to the right context with appropriate social and cultural conditions [24]. The context–mechanism–outcome (CMO) configuration is used as the main structure for the evaluation to identify contextual factors (features of the conditions that influence the mechanisms of interventions) and mechanisms (what and how components of interventions result in changes) that are associated with variation in outcomes [24,25]. This study protocol documents an on-going RE of ViP using a mixed method approach. Table 1 describes the initial underpinning programme theories related to ViP.

**Table 1:** Initial underpinning program theories.


IPT	IF	THEN	OUTCOME

1	If generalist nurses and doctors are trained with palliative care knowledge	Generalists will feel more confident in identifying cases and providing home PC	High acceptance, adoption and implementation fidelity of ViP

2	If advance care planning is conducted to support patients and caregivers in making informed decision related to care	Providers, patients and carers feel better prepared as there is an alignment for care at the end-of- life	High proportion of preferences honored at the end-of-life

3	If family carers are provided appropriate training and access to assistance to care for their loved ones at home	Carers will gain more confidence and feel more assured to provide care at home	Reduced caregiver burden and reduced acute hospital utilization

4	If a multidisciplinary team collaborate well through regular multidisciplinary meetings and open communication	Providers will work better with each other as they can trust each other to provide best care for patients and feel motivated to work towards a common goal of care	Adequate symptom management and appropriate coordination of services leading to reduced acute hospital utilization and healthcare cost

5	If guidelines and processes for provision of care under ViP is streamlined and implemented with high fidelity	Providers will be motivated to refer and participate in the provision of care with the common aim of providing best care for patients	Good quality of care

6	If there is sufficient support from key management and funding	Providers will feel supported and assured of the prospect of the program	Longer term sustainability


IPT: Initial program theory.

### Research Questions

Beyond answering if the program “works”, we propose to answer the following evaluation questions:

1. To what extent is ViP successfully implemented?
2. To what extent is ViP effective in achieving its intended outcomes?
3. What are the essential conditions for achieving program intended outcomes?
4. Whom (which population) does the program (not) work for and why?
5. How does ViP work to achieve its intended outcomes?

## Methodology

**Figure 2:**
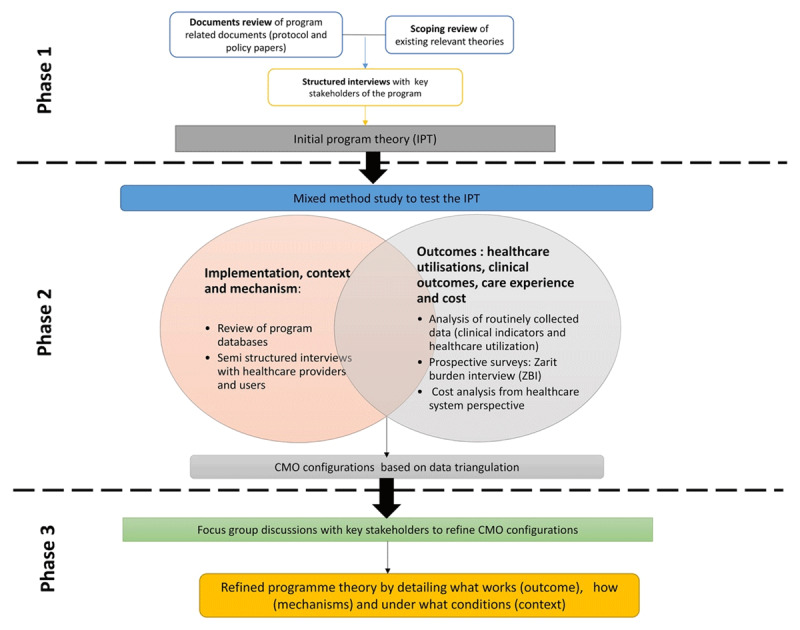
Realist Evaluation Phases of ViP.

According to the framework for RE outlined by Pawson and Tilley [24], evaluation will be conducted in three phases (Figure 2): (1) development of IPT, (2) testing of program theory using empirical data, and (3) refinement of IPT.

### Phase 1: Development of IPT – 6 months

Program related documents will be reviewed and a scoping review of reviews will be conducted. The JBI methodology for scoping review and PRISMA-ScR reporting guideline will be used [26]. The scoping review of reviews aims to identify existing theories related to (i) capacity building among generalists in the community and (ii) formation of integrated PC team for non-cancer patients. Relevant English language articles published since 2010 will be searched through Cochrane Library, Pubmed, CINAHL, and EMBASE. Documents and literature will then be assessed and data will be extracted guided by the RAMESES quality standards for realist synthesis [27] to derive initial program theories.

Concurrently, semi-structured interviews with key stakeholders will be conducted to assess initial program theories, perceived roles of ACP, caregiver support, capacity building of generalists and formation of integrated PC team. A purposive sampling of approximately 10 key stakeholders who were involved in the conceptualization of the program including program director, key medical providers and planning team will be interviewed.

Interviews will be recorded, transcribed verbatim and each transcript will be checked against recording for accuracy. Then, interview transcripts will be coded and analyzed by 2 evaluators using NVivo as suggested by Gilmore et al [28]. Transcripts will be entered into NVivo and themes will be coded as “node”. Transcripts will be reviewed, coded into relevant nodes and CMO configurations will be elicited from each node with Memo. CMO configurations are going to be continuously refine throughout the coding process. To ensure accurate representation of the IPT, initial CMO configurations will be circulated to stakeholders who were previously engaged for further input. After which, refinement to the IPT will be made before they are tested in phase 2.

### Phase 2: Testing of Program Theory using Empirical Data – 18 months

#### Study Design

A convergent parallel mixed methods study based on the triangulation design will be undertaken. Using the triangulation strategy, both quantitative and qualitative data will be collected concurrently. Data streams will be given equal weight and two datasets will be analyzed, compared and merged through iterative cycles of validation and confirmation of findings. Table 2 proposes a data framework which will be used to guide data collection according to the evaluation questions. Depending on the IPT elicited in phase 1, data framework will be revised accordingly.

**Table 2:** Data Framework for Data Collection.


IPT	CONTEXT			MECHANISM				OUTCOME			
		
	INDICATORS	DATA SOURCE	MEASUREMENT MOMENT	INDICATORS	DATA SOURCE	MEASUREMENT MOMENT	SAMPLE SIZE	INDICATORS	DATA SOURCE	MEASUREMENT MOMENT	SAMPLE SIZE

1	No. of generalist nurses and doctors who are trained with palliative care knowledge	Administrative database	Quarterly for the first 2 years of the programme	Confidence of generalists who were trained and completed training for ViP	Interviews with healthcare providers	At least 6 months after program implementation	15/group	Perception among implementation stakeholders (providers and users) about the acceptability of the program	Interviews with healthcare providers & users	At least 6 months after program implementation	15/group

								Program acceptance rate (% of participants correctly identified in reference to all referrals)	Administrative database	Quarterly for the first 2 years of programme	Enrolees in year 1 and 2

								Dropout rate (% of eligible participants who were initially enrolled but withdrew from the programme)			

								Timeliness of care (% of patients with their palliative care commence within 2 days after when they are deemed to be “ready for care”)			

								Responsiveness to urgent needs (% of patients unstable for ≤ 3 days)			
			
2	% of enrolled patients with ACP reviewed or completed		Quarterly for the first 2 years of the programme	Patients’ and/or caregivers’ experience with ViP	Interviews with caregivers	At least 2 encounters with ViP team and <3 months after patient died		Concordance between documented treatment preference and actual treatment and place of death		At death	
	
3	% of needy patients who were provided support	Administrative database & interviews with caregivers	Quarterly for the first 2 years of the programme					Change in caregiver burden score before and after intervention	Zarit Burden Interview-12	Baseline (at enrolment) and every 3 months after enrolmentuntil patients’ death	

								No. of ED attendances, in-patient admission and length of stay (LOS)	Administrative databases	1 month (30 days), 3 months (90 days) and 6 months (180 days) prior to death	

								% of patients with good management of symptoms	PCOC – symptoms assessment		
	
4	% of cases discussed in the MDM (multidisciplinary meetings)	Administrative database		Providers’ working experience	Interviews with providers	At least 6 months after program implementation		Development costs, program implementation costs and healthcare utilization costs	Administrative database	6 months prior to death	
		
	Quality of collaborative and communications between providers	Interviews with providers						Average duration in each PCOC stage		1 month (30 days), 3 months (90 days) and 6 months (180 days) prior to death	
	
5	Implementation experience	Interviews with providers	At least 6 months after program implementation					Perceived quality of care	Mortality follow-back survey	≤3 months after death	
	
*6*	Extent of support from management							Sustainability	Clinical Sustainability Assessment Tool (CSAT)	At least 6 months after program implementation	


#### Study participants

Two distinct groups of study participants – healthcare providers/managers and healthcare users will be recruited into this study. Healthcare providers/managers who are involved in the implementation of ViP will be invited to participate in the interviews through emails. Healthcare users will be recruited through ViP team/physicians in-charge and/or home care nurses.

#### Data Collection

Context, mechanisms and relationships between context and outcomes, as defined in phase 1, will be tested through interviews with healthcare providers and users. As suggested by Manzano [29], questions to be asked in these interviews aim to ascertained how the program works, for whom, in what circumstances and how implementation has veered from what was initially planned. A purposive sample of 15 healthcare providers/managers and 15 healthcare users (patient and/or carers) per disease condition will be invited to take part in face-to-face or virtual interviews. The eventual sample size will be determined based on thematic saturation of the study. Data saturation is reached when the ability to obtain additional new information has been attained, and when further coding is no longer feasible [30].

In ensuring the quality of data collected, an interview guide will be used and interviewers will routinely reflect on interview experience and discuss data as they are being collected. Based on emerging understanding and identification of key knowledge gaps throughout the process of interviewing, interview questions will be revised accordingly.

Healthcare services utilization, health outcomes, healthcare cost and caregiver burden data will be collected and analyzed over time to evaluate intervention outcomes of ViP. A quasi-experimental design will be adopted by comparing the outcomes of the intervention group against a comparator group for healthcare utilization, outcomes and cost. Due to limited data available, a pre-post comparison will be made for caregiver burden. A quasi-experimental design is selected as it more truly mimics real-world constraints and provide a balance between internal and external validity [31]. Pragmatically, all patients who agree to participate, are accepted into the home-based PC service, utilized the services for at least one month and passed away as of December 2022 will be included as the intervention group. A retrospective comparator group will be identified from available CGH and SACH clinical databases. We plan to restrict the databases to within CGH and SACH for the identification of comparators. This is because patients are planned to be recruited from CGH and SACH in the first 2 years. This is a way of ensuring comparability between intervention and comparator group while keeping the data manageable.

In identifying retrospective comparator group, relevant databases will initially be filtered for individuals who were referred to home hospice services between 2016–2019, have completed their advance care planning (ACP) and have died as of December 2020. Death status will be obtained from National Registry of Diseases Office records. Then, using a propensity matching strategy, a comparator group matched to intervention group in terms of age, gender, ethnicity, functional status, clinical status and re-admission characteristics will be identified. A ratio of 1:1 of intervention to comparator group will be used for matching.

In the assessment of healthcare utilization and health outcomes, date of death will be used as reference point for comparison. Acute hospital utilization (emergency department (ED) attendances, inpatient admissions, intensive care unit (ICU) admissions and length of stay (LOS)) will be extracted backwards from the date of death for 1 month (30 days), 3 months (90 days) and 6 months (180 days). Concordance of actualized place of death with preferences discussed as part of ACP will be determined after death as described by Tan et al [32].

We take a healthcare system perspective in examining the effect of ViP on healthcare cost. Development costs, program implementation costs and healthcare utilization costs will be measured. Development costs include costs incurred during the development of ViP. The program implementation costs consider costs related to the implementation of the program including the manpower, travel, equipment, materials used for patient and caregiver training and costs associated with multidisciplinary team meetings. The development and implementation costs will be systematically collected using the WHO’s CostIt instrument [33] and will be divided by the number of clients enrolled into the program to obtain per patient cost. Healthcare utilization cost for the use of hospital inpatient and outpatient services and primary care services in the metrics of full gross bill amounts will be extracted for six-month period before death.

Data for both intervention and comparator group will be extracted from SingHealth-iHiS Electronic Health Intelligence System (eHints), a common database of patient level data across all SingHealth institutions. Development and implementation cost will be extracted from ViP program database and potentially finance department. For comprehensiveness, we may also merge dataset with data held by the Ministry of Health to obtain nationwide acute hospital utilization (ED visits, hospital admissions, ICU utilizations, length of stay and date of death) using National Registration Identification Card (NRIC) of subjects included in the study.

Primary informal caregivers of ViP patients will be invited to complete a Zarit Burden Interview (ZBI) – 12 items to assess caregiver burden at enrolment and every 3 months after enrolment until patients’ death. The ZBI is a widely used tool for assessing caregiver burden among those who provide care for patients with various illnesses, including those at the end-of-life [34,35]. A higher score on the ZBI represents higher burden. As suggested by Stagg and Larner, a score of “0–20”, “21–40” and “>40” will be considered as little or no, mild/moderate and high caregiver burden respectively [36].

## Data Analysis

Interviews will be transcribed verbatim and each transcript will be checked against recordings for accuracy. Interview transcripts and observation notes will be coded using NVivo. Interviews conducted in languages other than English will be translated and transcribed in English. Like phase 1, interview transcripts will be coded and analyzed by 2 evaluators using NVivo as suggested by Gilmore et al [28]. To ensure the rigor of data analysis, regular meetings will be planned among evaluators to discuss the emerging themes identified in the data. In addition, re-analysis of qualitative data will be conducted if discrepancy is identified. An objective third person will be called in if the discrepancy cannot be resolved between the two evaluators.

Quantitative data analysis will be conducted using STATA. First, univariate analysis will be conducted using chi-square for categorical variables and t-test/ANOVA for continuous variables. Then, to estimate the effect of the program on the outcome of interest, multivariable regression analysis (Poisson or Negative Binominal regression) will be used for count data and generalized linear models will be used for continuous data. For dichotomous outcome variables, logistic regression will be used. Subgroup analysis may be conducted by patients’ primary conditions to allow for the detection of differential effects of ViP on various disease groups. To ensure robustness of the analysis, sensitivity analysis will also be performed.

### Data Triangulation

After both quantitative and qualitative data have been analyzed separately, findings will be integrated through a triangulation process at interpretation stage. The triangulation protocol [37] will be adopted to guide data integration by first producing a convergence coding matrix according to the guiding conceptual frameworks to display findings emerging from each component followed by consideration of where there is agreement, partial agreement, silence or dissonance between findings from different data sources. Assessment of the fit of data integration will be conducted by examining the coherence of findings from various methods used, as suggested by Fetters et al. [38]. Data on contexts, mechanisms and outcomes will then be linked according to the RE CMO configuration through a process of juxtaposing in which findings on contexts and mechanisms will be used to explain outcomes observed. Subsequently, interpretation will be shared with the research team for scrutiny, discussion and verification.

### Phase 3: Refinement of IPT – 6 months

A few potential CMO configurations will be proposed. Stakeholder participants of phase 2 will be invited back for focus group discussions (FGD) to validate and ensure program evolution has been accounted for. The IPTs will be refined to highlight how to improve ViP by detailing what works (outcome), as well as how (mechanisms) and under what conditions (contexts).

## Discussion

This protocol describes a RE of a home-based PC program for non-cancer patients in Singapore. RE is adopted to provide an in-depth understanding about whether the program works, for whom and in what circumstances. This study carries significant implications for various stakeholders. For the program, the study will generate new insights about the implementation, effectiveness of the program and explicit context-specific recommendations. By understanding what works and does not work, relevant stakeholders can refine program components and focus resources to ensure effectiveness of the program. Information on whom ViP works will help to refine selection criteria. Meanwhile, insights on mechanisms and contextual influence on ViP may be useful in informing required adaptations in the program strategies.

For policy makers, findings from the study will inform the potential of generalist involvement in home-based PC and policy-relevant insights for future enhancement of PC to support the aging population. Globally, most prior evaluations of home PC programs [11,39,40,41,42] focused mainly on assessing the effectiveness with limited information on the implementation processes. Also, few of these prior studies reported the use of RE approach. This study findings can be used to illustrate the applicability/suitability of the realist approach in the field of PC.

## Strengths and Limitations

The RE approach adopted in this study provides a useful guiding framework for a comprehensive evaluation of ViP. It is well fitted as RE seeks to answer relevant questions of interest, is flexible and able to accommodate changes surrounding interventions implemented in real-world setting. Moreover, the use of mixed methods allow us to draw on the strengths of both qualitative and quantitative methods, enhancing the credibility of the evaluation findings and allows for generation of in-depth insights.

With this evaluation approach, evaluators work closely with the key stakeholders (program designers, implementers, patients and caregivers) throughout the evaluation journey to co-define evaluation objectives, IPT, identify constraints to the feasibility of implementation of the evaluation itself, collect data, interpret findings and refine program theories. This provides confidence on the relevance of the evaluation efforts and likelihood for adoption of recommendations to improve relevant components of ViP and/or define future PC models.

Nevertheless, RE approach and mixed methods design are relatively new in comparison to the positivist approach commonly used in the evaluation of medical interventions. This may draw criticism for not being as rigorous as the typical RCT due to the lack of knowledge on the utility of this niche approach. Furthermore, we acknowledge that the use of mixed methods design also increases complexity. We strive to reach optimal integration of data at multiple levels – study design, methods, interpretation and reporting – using the convergent parallel mixed methods approach which connects and merges methods and findings [38].

Furthermore, as participation in the evaluation efforts is voluntary, we also recognize that selection bias may be introduced in this study. To account for selection bias, demographic information including age, gender, ethnicity and role (for healthcare providers) are collected and will be compared between study participants and those who declined to participate. Given the sensitivity around the topic of PC and prospective data collection methods, difficulties in recruitment and follow-up with respondents are also anticipated. In order to maximize recruitment numbers, evaluators will work closely with main care providers to obtain “buy-in”. If necessary, the recruitment period will also be extended to allow more people to participate. To minimize the numbers lost to follow-up, research appointments are scheduled at participants’ time and place of convenience in collaboration with participants’ healthcare provider. Where loss to follow-up inevitable due to death, survival analysis will be conducted to account for the missing data.

## Conclusion

Using the proposed methodology, the planned evaluation will highlight what works (outcomes), how (mechanisms) and for whom under what conditions (contexts) related to ViP. Findings gathered from this study will shed insights to inform decisions about implementation and further development of ViP. Furthermore, application of realist methodology for evaluation of complex interventions will also be illustrated.
